# α-Fetoprotein-Producing Endometrial Carcinoma Is Associated With Fetal Gut-Like and/or Hepatoid Morphology, Lymphovascular Infiltration, *TP53* Abnormalities, and Poor Prognosis: Five Cases and Literature Review

**DOI:** 10.3389/fmed.2021.799163

**Published:** 2021-12-15

**Authors:** Tomoyuki Otani, Kosuke Murakami, Naoki Shiraishi, Man Hagiyama, Takao Satou, Mitsuru Matsuki, Noriomi Matsumura, Akihiko Ito

**Affiliations:** ^1^Department of Pathology, Kindai University Faculty of Medicine, Osaka-Sayama, Japan; ^2^Division of Hospital Pathology, Kindai University Hospital, Osaka-Sayama, Japan; ^3^Department of Obstetrics and Gynecology, Kindai University Faculty of Medicine, Osaka-Sayama, Japan; ^4^Genome Medical Center, Kindai University Hospital, Osaka-Sayama, Japan; ^5^Department of Radiology, Kindai University Faculty of Medicine, Osaka-Sayama, Japan

**Keywords:** endometrial cancer, uterine corpus, alpha-fetoprotein (AFP), fetal gut-like, enteroblastic, hepatoid

## Abstract

The clinicopathological, immunohistochemical, and molecular characteristics of α-fetoprotein (AFP)-producing endometrial carcinoma (AFP+ EC) are poorly understood. From 284 cases of endometrial carcinoma in our pathology archive, we identified five cases (1.8%) of AFP+ EC with fetal gut–like (4/5) and/or hepatoid (2/5) morphology. All cases exhibited lymphovascular infiltration. In addition, 24 cases of endometrial carcinoma with elevated serum AFP levels were retrieved from the literature. The patient age ranged from 44 to 86 years (median: 63). Of 26 cases whose FIGO (International Federation of Gynecology and Obstetrics) stage and follow-up information was available (mean follow-up 24 months), 15 were stage I or II and 11 were stage III or IV. Even in stage I or II disease, death or relapse occurred in more than half of the patients (8/15). Detailed analysis of our five cases revealed that, on immunohistochemistry, AFP+ EC was positive for SALL4 (4/5), AFP (3/5), and HNF1β (4/5) in >50% of neoplastic cells and negative for estrogen and progesterone receptors (5/5), PAX8 (4/5), and napsin A (5/5). Four cases exhibited aberrant p53 immunohistochemistry and were confirmed to harbor *TP53* mutations by direct sequencing. No mutation was found in *POLE, CTNNB1*, or *KRAS*. In conclusion, AFP+ EC merits recognition as a distinct subtype of endometrial carcinoma, which occurs in 1.8% of endometrial carcinoma cases, are associated with *TP53* abnormalities, exhibit lymphovascular infiltration, and can show distant metastasis even when treated in early stage.

## Introduction

Alpha-fetoprotein (AFP), an oncofetal protein that is physiologically produced by the liver, yolk sac, and gut during the prenatal period (1), is known to be produced in specific types of tumors. Hepatocellular carcinoma in adults and yolk sac tumor, a subtype of germ cell tumor, in children and young adults are the two most famous examples. Curiously, a small subset of carcinomas of the stomach (2, 3) and lung (4, 5) is also associated with elevated serum AFP levels and these relatively rare carcinomas exhibit histomorphological resemblance to hepatocellular carcinoma or immature endoderm-derived structures, such as the fetal gut or fetal lung. In the endometrium, AFP-producing neoplasms are rare. These include hepatoid carcinoma, unspecified adenocarcinoma with AFP production, and yolk sac tumor. AFP-producing endometrial carcinoma (AFP+ EC) (6–28), which can be defined to include endometrial hepatoid carcinoma (10–16, 19–21, 24–28) and adenocarcinoma with AFP production (6–9, 17, 18, 22, 23), are known only in scattered case reports. Some of them are reported to coexist with conventional (i.e., Müllerian) endometrial carcinoma or carcinosarcoma (11–16, 19–21, 24–26). A few reports have characterized their cases of AFP-producing adenocarcinoma as endometrioid carcinoma (18, 22), though relevant immunohistochemistry (IHC) is rarely reported.

Endometrial yolk sac tumor is somewhat better characterized and reported in a few case series, often together with ovarian examples and/or other germ cell tumors (29–33). Especially in postmenopausal women, endometrial yolk sac tumors also often coexist with Müllerian carcinoma or carcinosarcoma. The yolk sac tumor component in these mixed tumors is interpreted as “retrodifferentiation” (34) of Müllerian carcinoma and sometimes designated as “somatically derived yolk sac tumor” (29, 30). Approximately half of yolk sac tumors of the endometrium in older patients do not show characteristic features of the tumors of the same name in young patients, such as microcystic and reticular architecture, loose myxoid stroma, or Schiller-Duval bodies, and instead exhibit predominantly glandular and papillary architecture. Therefore, at least some of these “glandular yolk sac tumors” (32) can be regarded as the same tumors that are reported under the name of AFP+ EC.

In this study, we reviewed cases of AFP+ EC reported in the literature and performed detailed pathological, immunohistochemical, and molecular analyses of five cases from our pathology archive.

## Materials and Methods

### Case Selection

Two cases of AFP+ ECs were diagnosed during the routine diagnostic practice at Kindai University Hospital, Osaka-Sayama, Japan. To search for additional cases, all cases of endometrial neoplasms that underwent hysterectomy at Kindai University Hospital between 2015 and 2020 were identified and pathology slides were retrieved from the pathology archive. All available slides were reviewed. In this part of the study, AFP+ EC was defined as an endometrial neoplasm bearing a sufficient morphological and immunohistochemical resemblance to AFP-producing carcinomas of the stomach and lung, at least in some part of the tumor. AFP-producing carcinomas of the stomach and lung with characteristic histomorphology include gastric carcinoma with enteroblastic differentiation, fetal adenocarcinoma of the lung, and hepatoid adenocarcinoma of both organs. Morphologically suggestive cases were stained for AFP, SALL4, PAX8, and CK7 to aid in the diagnosis. To be included as AFP+ EC in this part of the study, AFP and, given the high sensitivity of this marker for AFP-producing carcinomas of the stomach and lung, SALL4 were required to be positive in ≥ 1% of tumor cells. PAX8 and CK7 were used as Müllerian markers. Serum AFP levels were not available at the time of histological examination. Two pathologists (TO and AI) reviewed the candidate cases and identified three additional cases of AFP+ EC. We also searched for endometrial yolk sac tumors with typical morphology as seen in young patients. In total, five cases of AFP+ ECs were included in the study. Clinical data were obtained from medical records. This study was approved by the Institutional Review Board of Kindai University Faculty of Medicine (R02-311).

**Figure 1:**
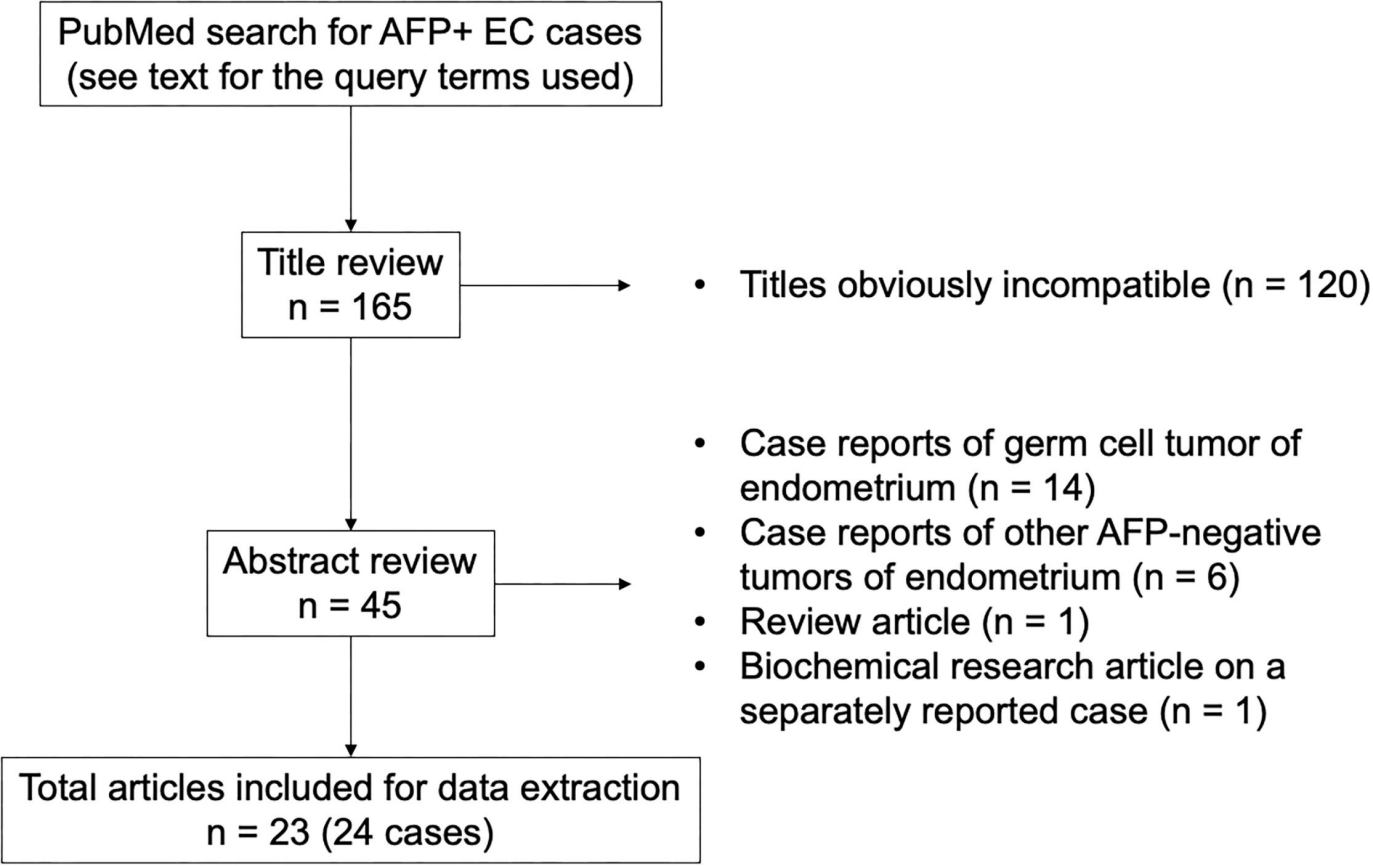
Selection of reported cases.

Additional cases were searched in the PubMed database (Figure 1). The query terms were “(alpha-fetoprotein OR AFP) AND (carcinoma OR adenocarcinoma OR carcinosarcoma OR müllerian) AND (endometrium OR endometrial OR uterus OR uterine)” and “hepatoid AND (endometrium OR endometrial OR uterus OR uterine).” The search with these terms retrieved 159 and 38 articles written in English, respectively, which amounted to 165 articles after excluding the duplicate results. Based on the title and abstract, 23 articles were considered for inclusion. Since a detailed histological review was impossible with these reported cases, all cases of endometrial tumors with elevated serum AFP levels histologically described as carcinoma or carcinosarcoma (but not yolk sac tumor) were included. Hepatoid carcinoma was considered as carcinoma and not a variant of yolk sac tumor for the purpose of this study. Our intention was to include all cases of endometrial hepatoid carcinoma regardless of serum AFP levels, but all reported cases of endometrial hepatoid carcinoma were associated with elevated serum AFP levels. Eventually, 24 cases of endometrial carcinoma and carcinosarcoma with AFP production from 23 articles were selected as AFP+ EC for our case review.

### Immunohistochemistry

IHC was performed on 4-μm-thick formalin-fixed paraffin-embedded (FFPE) tissue sections. Antibodies and their clones, sources, and dilutions are presented in Supplementary Table 1. D2-40 and CD31 IHC were used to aid in the identification of vascular invasion. Immunostaining for p53 (35) and mismatch repair proteins (MLH1, PMS2, MSH2, and MSH6) (36) were interpreted according to previous reports. HER2 immunostaining was scored according to a previous study (NCT01367002) (37). Staining results were interpreted by two pathologists (TO and AI) independently, and in case of discrepancy, a consensus was achieved using a multiheaded microscope.

### Molecular Analysis for *TP53, POLE, CTNNB1, KRAS*, and *PIK3CA*

Tumor tissue was macrodissected from 10-μm-thick FFPE tissue sections to increase tumor purity. Genomic DNA was extracted using the QIAamp DNA FFPE Tissue Kit (Qiagen, Hilden, Germany) according to the manufacturer's instructions. PCR was performed with KOD FX Neo (TOYOBO, Osaka, Japan) (38) and the products were subjected to 2% agarose gel electrophoresis and sequenced using a 3500xL Genetic Analyzer (Applied Biosystems, Foster City, CA). For *TP53, POLE*, and *CTNNB1*, primers were designed using primer-BLAST (NCBI). Primers for *TP53* and *POLE* were designed to amplify exons 4-8 and exons 9, 13, and 14, respectively. Primers for *CTNNB1* were designed to amplify the region containing the mutation hotspots in exon 3. Primer sequences have been previously reported for *KRAS* exon 2 (39) and *PIK3CA* exons 10 and 21 (40). The primer sequences used in this study are listed in Supplementary Table 2.

Since it was difficult to histologically determine whether the metastatic lesions originated from an endometrial neoplasm or breast carcinoma in case 1, the genetic mutation detected in endometrial neoplasm was searched for in the tissue of the bone metastasis using the method described above.

## Results

### Clinical Characteristics

From 284 cases of endometrial carcinoma that had undergone hysterectomy at Kindai University Hospital between January 2015 and December 2020, five cases of AFP+ ECs were identified. No case was diagnosed as endometrial yolk sac tumor during this period. The clinical characteristics of these five cases are shown in Table 1. Additional 24 cases were identified in the English literature via a PubMed search and are summarized in Table 2. Overall, 29 cases of AFP+ EC were available for our analysis. The patient age ranged from 44 to 86 years (median, 63). Of the 26 cases whose FIGO (International Federation of Gynecology and Obstetrics) stage was available, 12 were stage I, 3 were stage II, and 11 were stage III or IV. The serum AFP levels were elevated in all the cases retrieved from the literature. Among the five cases from our institution, the serum AFP levels were elevated in two cases measured in patients with disease (cases 3 and 5), were within normal limits in two cases measured in a disease-free state (cases 2 and 4), and could not be evaluated in one case (case 1, archival case). Pre- or perioperative values were not available in these cases. Of note, case 3 had a normal serum AFP level measured in the disease-free state after surgery, but it increased during the observation period, and lung metastasis was eventually discovered.

**Table 1:** Clinicopathological characteristics of AFP-producing endometrial carcinoma.

**Case**	**Age**	**Gravidity/** **parity**	**Sx**	**Serum AFP (ref < 10 ng/mL)**	**FIGO stage**	**Metastasis**	**Surgery**	**Postoperative therapy**	**Follow-up**	**Gross findings**	**AFP+ component**	**Other component**	**Myometrial invasion**	**LVI^c^**
1	63	3/3	AVB	N/A	IA	Liver, lungs, bone (post surgery)	THBSO, LND	TC	DOD 11 Mo	28-mm nodular lesion associated with endometrial polyp	Fetal gut-like	CS^b^	Little	Present
2	73	2/2	AVB	WNL in disease-free state	IA	None	THBSO, LND	TC; RTx for Rec	Rec 17 Mo NED 32 Mo	27-mm lobulated polypoid mass with myometrial invasion	Hepatoid, fetal gut-like	CS^b^	< 1/2	Present
3	76	3/3	AVB	96 ng/mL at Rec	IB	Lungs (post surgery)	THBSO, LND	TC	Rec 24 Mo AWD 24 Mo	52-mm polypoid mass with myometrial invasion	Hepatoid, non-clear glandular	–	> 1/2	Extensive
4	48	0/0	AVB	WNL in disease-free state	IVB	LN	THBSO^a^, LND	TC	NED 43 Mo	11-cm uterus containing necrotic material, adhering to large and small intestines	Fetal gut-like, non-clear glandular	–	Extensive	Present
5	78	2/2	AVB	1,335 ng/mL 5 Mo post surgery	IVB	LN, bone	THBSO	TC	AWD 12 Mo	8-cm uterus containing necrotic material	Fetal gut-like, non-clear glandular	–	Extensive	Extensive

*AFP, α-fetoprotein; AVB, abnormal vaginal bleeding; AWD, alive with disease; CS, carcinosarcoma; FIGO, International Federation of Gynecology and Obstetrics; LN, lymph nodes; LND, pelvic and para-aortic lymphadenectomy; LVI, lymphovascular invasion; Mo, months; N/A, not available; NED, no evidence of disease; Rec, recurrence; RTx, radiation therapy; SO, salpingo-oophorectomy; Sx, presenting symptom; TC, paclitaxel and carboplatin; THBSO, total hysterectomy and bilateral salpingo-oophorectomy; WNL, within normal limits*.

a *Sections of large and small intestines attached to the uterus were also resected*.

b *Associated with endometrial polyp*.

c *Investigated with D2-40 and CD31 immunohistochemistry*.

**Table 2:** Reported cases of AFP-producing endometrial carcinoma.

**References**	**Age**	**Sx**	**Serum AFP (ng/mL)^b^**	**Histology as reported**	**AFP IHC**	**p53 IHC**	**Myometrial invasion**	**LVI**	**FIGO stage^c^**	**Metastasis**	**Follow-up**
Kawagoe (6)	65	AVB	1,476	CS	+	NR	2/3	NR	III	LN	NED 3 Mo
Matsukuma and Tsukamoto (7)	63	NR	670	Adeno	+	NR	slight	NR	I	LN, lung^e^	DOD 12 Mo
Kubo et al. (8)	55	AVB	676	Papillary adeno	+	NR	> 1/2	NR	III	Vagina, Douglas pouch; LN, liver, lung, bone^f^	DOD 3 Mo
Phillips et al. (9)	68^a^	NR	21,000^a^	CS	+	NR	NR	NR	I	NR	DOD 7 y^f^
Yamamoto et al. (10)	62	AVB	280.3	Hep, tubular adeno	+	NR	present	present	IV	Lung	DOD 3 Mo
Hoshida et al. (11)	66	AVB	16,170	Hep, EEC	+	NR	> 1/2	present	III	Uterine cervix, LN	DOD 32 Mo
Toyoda et al. (12)	60	AVB	31,950	Hep, EEC	+	NR	< 1/2	present	III	LN; lung^f^	DOD 12 Mo
Adams et al. (13)	66	AVB	351 on POD4	Hep, EEC	+	NR	1/2	NR	I	None^g^	NED 8 y
Takahashi and Inoue (14)	68	AVB	2,800	Hep, CS	+	NR	The tumor invaded the parametrium	present	NR	NR	NR
Takano et al. (15)	63	AVB	5,060	Hep, CS	+	NR	little or none	NR	I	None^g^	NED 12 Mo
Takeuchi et al. (16)	61	epigastric discomfort	453	Hep, EEC	+	NR	1/2	NR	IV	Omentum	NED 12 Mo
Tran et al. (17)	44	AVB	1,493 IU/mL	SC	+	+	NR	extensive	IV	Ov, sigmoid colon, diaphragm	NED 15 Mo
Kodama et al. (18)	59	abd swelling	1,292.8	EEC	+	NR	> 1/2	present	II	None	NED 60 Mo
Ishibashi et al. (19)	86	AVB	7,824	Hep, EEC	+	NR	slight	absent	I	LN^f^	Rec 11 Mo AWD 36 Mo
Hwang et al. (20)	75	AVB	90,508	Hep, EEC	+	NR	> 1/2	NR	I	None	NED 3 Mo
Kawaguchi et al. (21)	63	AVB	10,131	Hep, CS	+	NR	NR	NR	II	None	NED 2 y
Kawaguchi et al. (21)	82	AVB	401	Hep, CS	+	NR	> 1/2	NR	I	Lung^f^	DOD 1y
Akhavan et al. (22)	57	AVB	465.3	EEC	NR	NR	> 1/2	NR	II	Lung, skin, brain^f^	DOD < 1 y
Chen et al. (23)	65	AVB	244.8	CS	NR	NR	> 1/2	NR	NR	NR	NR
Wu et al. (24)	61	AVB	253.3	Hep, EEC	+	+	> 1/2	NR	III	LN; lung^f^	DOD 10 Mo
Kuroda et al. (25)	63	AVB	151	Hep, SC	+	+ in SC NR in Hep	< 1/2	NR	I	None	NED 2 Mo
Li et al. (26)	67	AVB	31,896	Hep, CS	+	NR	> 1/2	extensive	III	Ov; liver, peritoneum^f^	Rec 2 Mo DOD 11 Mo
Yin et al. (27)	64	AVB	3,931	Hep	+	NR	NR	NR	NR	NR	NR
Liu et al. (28)	48	AVB	1,210	Hep	+	–^d^	superficial	absent	I	None	NED 63 Mo

*abd, abdominal; adeno, adenocarcinoma; AFP, α-fetoprotein; AVB, abnormal vaginal bleeding; AWD, alive with disease; CS, carcinosarcoma; DOD, died of disease; EEC, endometrial endometrioid carcinoma; FIGO, International Federation of Gynecology and Obstetrics; Hep, hepatoid carcinoma; IHC, immunohistochemistry; LN, lymph nodes; LVI, lymphovascular invasion; NED, no evidence of disease; Mo, months; NR, not reported; Ov, ovaries; POD, postoperative day; Rec, recurrence; SC, serous carcinoma; Sx, presenting symptom; y, years*.

a *At recurrence*.

b *Pre-operative if not otherwise indicated*.

c *According to FIGO 2008*.

d *Not reported whether wildtype or null*.

e *At autopsy*.

f *Post surgery*.

g *Intraoperative peritoneal washing cytology was positive*.

Follow-up information was available from 26 cases, with a mean follow-up period of 24 months. Of the 11 patients with FIGO stage III or IV disease (mean follow-up, 14 months), 6 died of disease and 1 was alive with disease. Of the 15 patients with FIGO stage I or II disease (mean follow-up, 34 months), five died of disease and additional three suffered from recurrence; 4 of 7 patients with recurrence-free survival had a follow-up period of no more than 1 year.

### Histopathological Analysis

**Figure 2:**
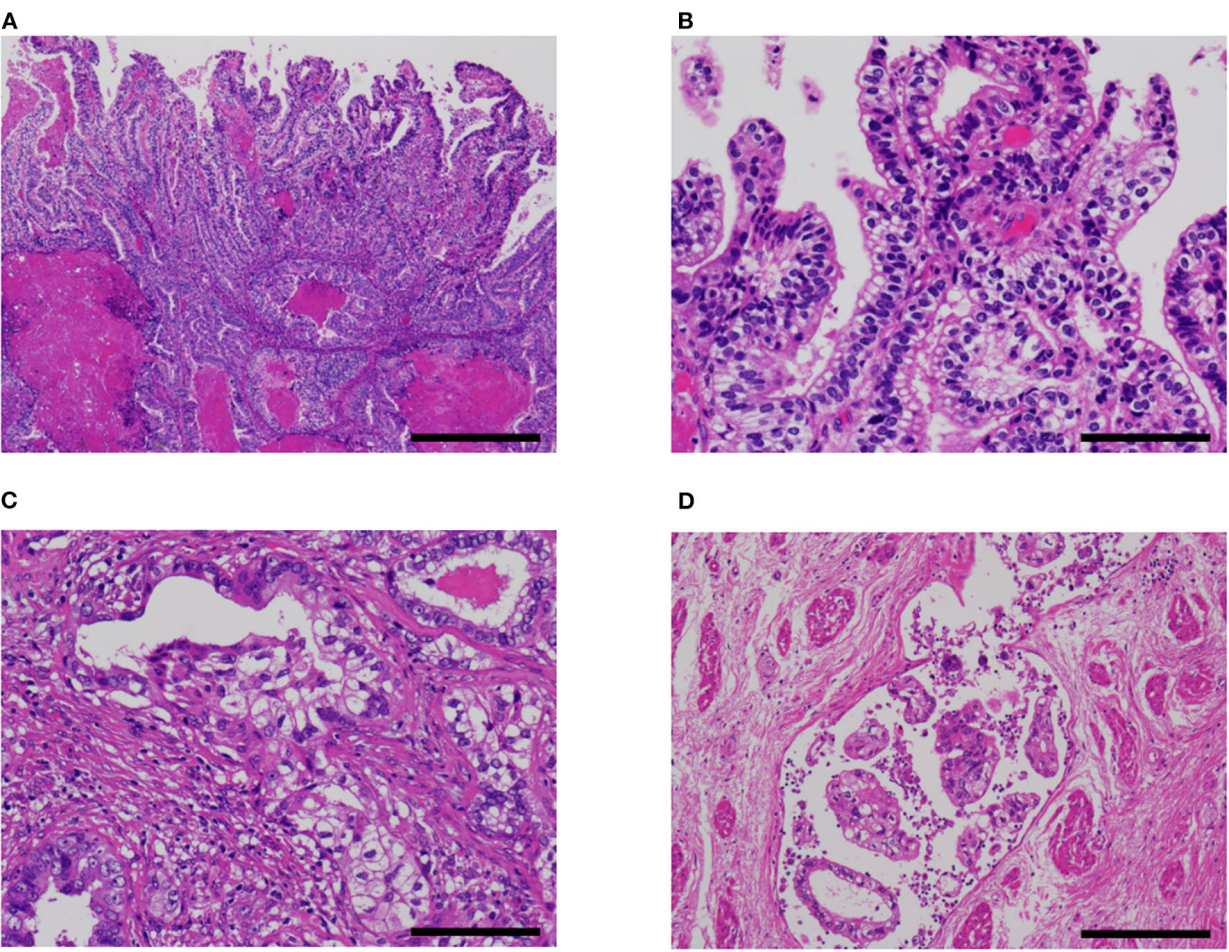
Fetal gut-like pattern of α-fetoprotein-producing endometrial carcinoma (case 5). **(A,B)** Fetal gut-like pattern was composed of tall columnar neoplastic cells with clear cytoplasms and large nuclei in papillary and glandular architecture. **(C)** Variable degrees of intraglandular piling-up of neoplastic cells could be seen and imparted clear cell carcinoma-like appearance. **(D)** In case 5, lymphovascular invasion was extensive. Hematoxylin-eosin; original magnification ×40 **(A)**, ×200 **(B,C)**, and ×100 **(D)**; 500 **(A)**, 100 **(B,C)**, and 200 μm **(D)**.

Pathological characteristics of our five cases are shown in Table 1. Four of the five cases had a fetal gut-like component (Figure 2). This component was composed of tall columnar cells with large nuclei and clear cytoplasms forming glandular and papillary structures, resembling carcinoma with enteroblastic differentiation of the stomach and fetal adenocarcinoma of the lung. When its apical border was smooth, this component had a superficial resemblance to the secretory variant of endometrioid carcinoma, with tall cells and optically clear cytoplasms (Figures 2A,B). In other parts, intraglandular piling-up of neoplastic cells in invasive glands gave them a near-solid, nest-like appearance and this was reminiscent of clear cell carcinoma (Figure 2C).

**Figure 3:**
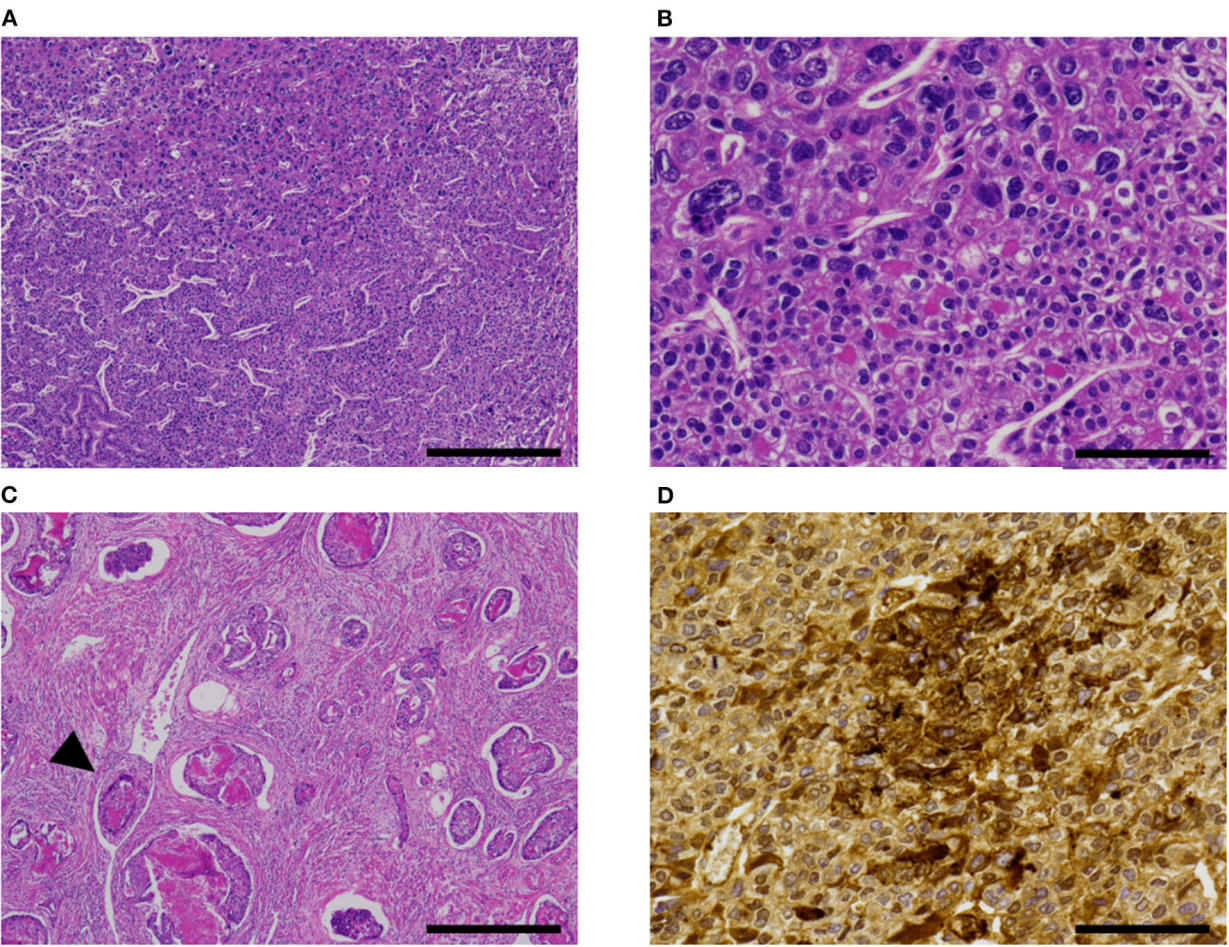
Hepatoid carcinoma (case 3). **(A)** This pattern was composed of tightly arranged trabecular neoplastic epithelium with intervening sinusoid-like capillaries. **(B)** Canaliculi-like small lumina were also observed. Tumor cell nuclei had coarse chromatin and showed moderate to severe nuclear atypia. **(C)** In case 3, this carcinoma exhibited extensive myoinvasion and vascular invasion (arrowhead; retraction clefts are also pictured). **(D)** Neoplastic cells were positive for AFP **(D)**. Hematoxylin-eosin **(A–C)**; original magnification ×40 **(A,C)** and ×200 **(B,D)**; scale bars = 500 μm **(A,C)** and 200 μm **(B,D)**. AFP, α-fetoprotein.

Hepatoid carcinoma, seen in two cases, was composed of tightly arranged trabecular neoplastic epithelium with intervening sinusoid-like capillaries (Figure 3). Between tumor cells, canaliculi-like structures could often be observed. The tumor cells had a moderate amount of eosinophilic or clear cytoplasms. Tumor cell nuclei had coarse chromatin and nuclear atypia was moderate to severe. In case 2, a gradual transition between the hepatoid carcinoma and fetal gut-like component was observed. Hepatoid carcinoma showed exophytic polypoid growth into the uterine cavity in both cases and also showed myoinvasion in one case (case 3, Figure 3C).

**Figure 4:**
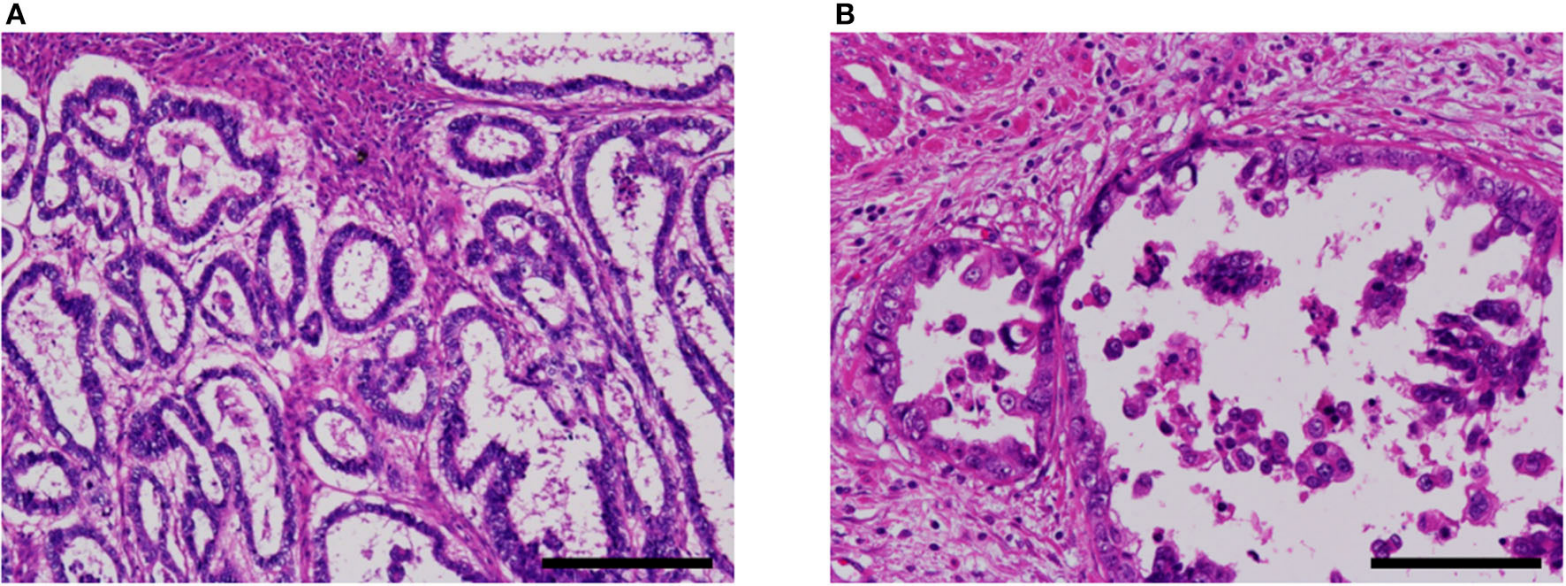
Non-clear glandular pattern of α-fetoprotein-producing endometrial carcinoma (case 5). **(A,B)** This pattern was associated with smooth **(A)** or ragged **(B)** luminal border, which imparted low-grade endometrioid-like or clear cell carcinoma-like appearance, respectively. Hematoxylin-eosin; original magnification ×100 **(A)** and ×200 **(B)**; scale bars = 200 μm **(A)** and 100 μm **(B)**.

A non-clear glandular component was observed in three cases (Figure 4). In two cases (cases 3 and 5), this component invaded the myometrium as discrete glandular structures. In case 4, the neoplastic epithelium formed confluent, anastomosing glands, which some observers might call reticular. The non-clear glandular pattern was low-grade endometrioid-like when the apical border was smooth (Figure 4A) and clear cell carcinoma-like when associated with hobnail-like nuclear protrusion (Figure 4B).

**Figure 5:**
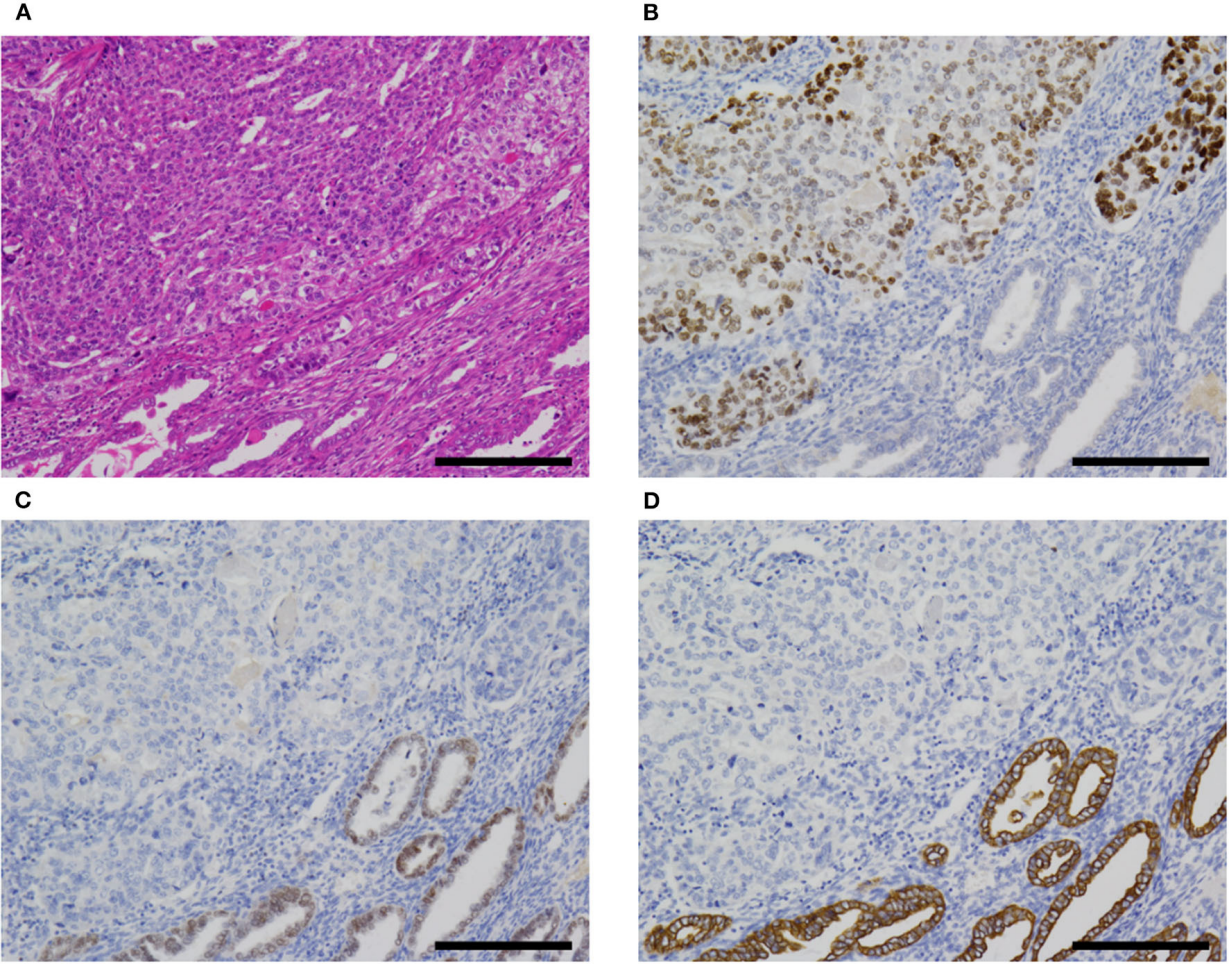
AFP+ EC associated with Müllerian component (case 2). **(A)** In cases 1 (not shown) and 2, AFP+ EC was associated with Müllerian carcinosarcoma (sarcomatous component not shown). **(B–D)** The former (upper left) was SALL4+ **(B)**, PAX8- **(C)**, and CK7- **(D)**, while the latter (lower right) was SALL4-, PAX8+, and CK7+. Hematoxylin-eosin **(A)**; original magnification ×100; scale bars = 200 μm. AFP+ EC, α-fetoprotein-producing endometrial carcinoma.

Two cases were associated with carcinosarcoma (cases 1 and 2; Figure 5). These two cases were also associated with an endometrial polyp. In both cases, the epithelial component was most consistent with serous carcinoma, at least in part, but some areas were difficult to classify and might represent a transition between AFP+ and Müllerian components.

All cases exhibited lymphovascular infiltration (Figures 2D, 3C).

### Immunohistochemistry and Molecular Analysis

**Figure 6:**
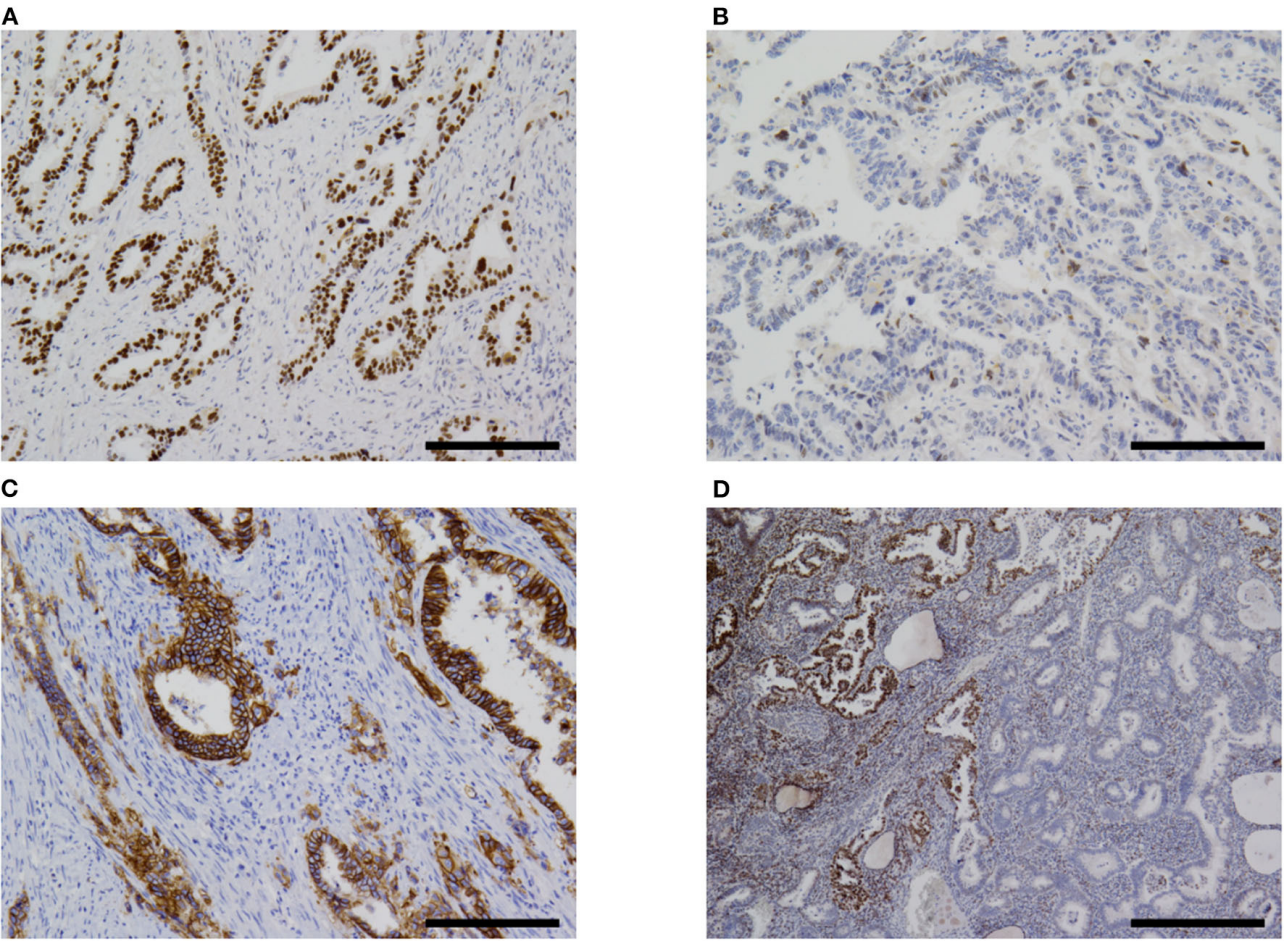
Immunohistochemistry. **(A)** Most cases of α-fetoprotein-producing endometrial carcinoma were p53-aberrant on immunohistochemistry (case 5 shown). **(B)** Only one case in our series was p53-wildtype (case 4). **(C)** Some overexpressed HER2 (case 5). **(D)** One case (case 2) in our series showed subclonal loss of expression of MLH1 and PMS2 (not shown). Original magnification× 100 **(A–C)** and ×40 **(D)**, scale bars = 200 μm **(A–C)** and 500 μm **(D)**.

The immunohistochemistry and molecular analysis results are shown in Table 3 and Figures 3D, 5, 6. The AFP-positive component of all the five cases was at least focally positive for AFP (Figure 3D) and SALL4 (Figure 5B). PAX8 (Figure 5C) and CK7 (Figure 5D) were never diffusely positive, although the epithelial component of coexisting carcinosarcoma in two cases stained diffusely with PAX8 and CK7 (Figures 5C,D). Estrogen and progesterone receptors were always negative in AFP-positive component. HNF1β was diffusely positive in the AFP-positive component at least weakly, whereas napsin A was always negative. p53 IHC was abnormal in four of the five cases (Figure 6A) and wild-type in the remaining case (Figure 6B). Two cases showed strong positive staining of > 80% of neoplastic cells (Figure 6A), while the other two cases with abnormal staining showed heterogeneous expression.

**Table 3:** Immunohistochemical and molecular analysis of AFP-producing endometrial carcinoma.

**Case**	**Immunohistochemistry**											**Molecular analysis**				
		**AFP**	**SALL4**	**PAX8**	**CK7**	**ER/PR**	**HNF1β**	**Napsin A**	**p53 IHC**	**MMR^e^**	**HER2**	***TP53***	***POLE***	***CTNNB1***	***KRAS***	***PIK3CA***
1	AFP+	1+	2+	-	3+^a^	-	4+	-	Aberrant^b^	Normal	2+	c.742C> T (R248W)	wt	wt	wt	wt
	AFP-	-	-	4+	4+	1+	-	-	Aberrant^c^	Normal	2+					
2	AFP+	3+	4+	-	-	-	4+	-	Aberrant^d^	Subclonal loss of MLH1^f^	1+	c.675_695delinsC (V225fs)	wt	wt	wt	c.3062A> G (Y1021C)
	AFP-	1+	1+	4+	4+	1+	2+	-	Aberrant^c^	Subclonal loss of MLH1^f^	2+					
3		3+	3+	-	1+^a^	-	2+	-	Aberrant^b^	Normal	1+	c.763A> T (I255F)	wt	wt	wt	wt
4		3+	3+	-	-	-	3+	-	wt	Normal	0	wt	wt	wt	wt	wt
5		1+	4+	1+	3+^a^	-	3+	-	Aberrant^b^	Normal	3+	c.376-3_376-1delinsT (splice acceptor site)	wt	wt	wt	c.1658_1659delinsC (S553fs)

*-, positive in < 1% of tumor cells; +, positive in 1–25% of tumor cells; 2+, positive in 26–50% of tumor cells; 3+, positive in 51–75% of tumor cells; 4+, positive in 76–100% of tumor cells; AFP, α-fetoprotein; AFP+, AFP-positive (fetal gut-like or hepatoid) component; AFP-, AFP-negative (Müllerian) epithelial component; CK, cytokeratin; ER, estrogen receptor; IHC, immunohistochemistry; PR, progesterone receptor; wt, wild-type*.

a *Mosaic-like*.

b *Diffuse (>80% tumor cells)*.

c *Heterogeneous*.

d *Null (no tumor cells)*.

e *Mismatch repair proteins (i.e., MLH1, PMS2, MSH2, and MSH6)*.

f *Only some tumor cells express MLH1 and PMS2; all tumor cells retain MSH2 and MSH6 expression*.

All the four cases with abnormal p53 IHC were found to harbor mutations in *TP53* gene. Two cases harbored mutations in exon 21 of *PIK3CA*. No mutations were found in *POLE* exons 9, 13, and 14, *CTNNB1* exon 3, *KRAS* exon2, or *PIK3CA* exon 10. In case 1, *TP53* c.742C> T mutation was detected in both endometrial and bone lesions.

## Discussion

AFP+ EC is a rare neoplasm. The 2020 WHO Classification of Female Geniatal Tumors (41) does not mention AFP+ EC or hepatoid carcinoma. Yolk sac tumor is described under the heading of “germ cell tumors of the uterine corpus.” Previous cases reported as AFP+ EC, excluding cases reported as yolk sac tumor, are summarized in Table 2.

Hepatoid carcinoma (Figure 3) has been recognized as a type of AFP+ EC since 1996 (10, 11). This histological type of endometrial carcinoma is often reported to exhibit exophytic growth into the uterine cavity grossly and vascular infiltration microscopically (Table 2) (10–16, 19–21, 24–28).

Fetal gut-like carcinoma (Figure 2) has not been recognized as such in endometrial carcinoma, as far as we are aware. In the previous literature, this pattern in endometrium has been reported as yolk sac tumor by some authors: Ravishankar et al. (30) reported 11 cases and Fadare et al. (32) reported 9 plus 3 cases of endometrial yolk sac tumors, mostly in postmenopausal patients. According to their reports, the histology of some examples was indistinguishable from yolk sac tumors in younger patients, while others showed exclusively glandular and papillary architecture. The latter histological pattern is consistent with what we termed the fetal gut-like pattern in this article. Fetal gut-like morphology is not uncommonly seen in AFP-producing carcinoma of the stomach and the lung: carcinomas with this pattern are called enteroblastic carcinoma in the stomach (2, 3) and fetal adenocarcinoma in the lung (4). In the gynecological tract, AFP-producing carcinomas of the ovary (42–44) and uterine cervix (45, 46) have been described to exhibit this morphology.

Three of our cases also contained a non-clear glandular component (Figure 4) showing the immunophenotype of AFP+ EC: namely, AFP+, SALL4+ and PAX8-. This component might simply be a fetal gut-like component with decreased cytoplasmic glycogen. Although difficult to distinguish from much more common Müllerian carcinoma by histomorphology alone (Figure 4), this immunohistochemical pattern is inconsistent with Müllerian carcinoma and we consider this non-clear glandular component to be an integral part of AFP+ EC. In theory, it is entirely conceivable that AFP+ EC composed exclusively of non-clear glandular component exists. Indeed, some of the previously reported AFP-producing “endometrioid” carcinomas (18, 22) might fit this description.

AFP+ EC is an aggressive neoplasm. About half of the cases in our series and the literature were diagnosed at FIGO stage III or IV. Although other cases were diagnosed earlier, these patients hardly fared better: 5 out of 15 patients with stage I or II disease succumbed to the disease and additional 3 experienced recurrence, while 6 out of 10 patients with stage III or IV disease died of disease. This aggressive behavior even in patients with early-stage disease is most likely related to the often extensive vascular infiltration of this neoplasm. In keeping with this hypothesis, disproportionately many cases of relapse in stage I or II patients manifested as distant metastasis (five out of seven cases with available information). As for cases reported as endometrial yolk sac tumor, which should contain at least some AFP+ ECs, the outcomes are somewhat mixed: Ravishankar et al. (30) reported poor outcomes comparable to our series, while cases reported by Fadare et al. (32) seem to have fared better.

The similarity between AFP-producing neoplasms of the endometrium and stomach is striking: they arise in older patients and can be associated with conventional adenocarcinoma; although typical yolk sac tumor morphology with reticular and microcystic pattern can occur, fetal gut-like pattern (carcinoma with enteroblastic differentiation in stomach and fetal gut-like carcinoma/glandular yolk sac tumor in endometrium) seems to be more frequent (47); fetal gut-like neoplasm and hepatoid carcinoma often coexist; hepatoid carcinoma grows exophytically into the lumen; they are associated with *TP53* mutation, vascular infiltration, and hematogenous metastasis (2, 3). Indeed, hepatoid carcinoma of the endometrium was at first described as an endometrial carcinoma analogous to hepatoid carcinoma of the stomach (24, 27). Some authors propose to call all these AFP-producing neoplasms associated with conventional carcinoma in various organs with the name of “somatically derived yolk sac tumor” (29).

HER2 is reported to be positive in 30% of endometrial serous carcinomas (37). The use of anti-HER2 therapy in these cases was recently described in the National Comprehensive Cancer Network guidelines (48). HER2 is overexpressed in many AFP-producing gastric carcinomas (49) and a case of fetal gut-like adenocarcinoma of the uterine cervix with amplified and overexpressed HER2 has been reported (46). We hypothesized that AFP+ EC might also be associated with HER2 overexpression, but the frequency of HER2 positivity by immunohistochemistry was not significantly different from that reported for serous carcinoma (50). However, our number of cases is small and the possibility remains that further accumulation of cases might reveal an association between AFP+ EC and HER2 overexpression and/or amplification.

In addition to prognostication and possible therapeutic impact, another important reason to recognize AFP+ EC is the post-operative surveillance: serum AFP can be a useful tumor marker for patients with this disease. Our case 3 is a case in point. Although serum AFP levels were not available from pre- or perioperative period, this patient was followed up with serum AFP after postsurgical pathological examination demonstrated that the tumor cells were immunohistochemically positive for AFP. Serum AFP levels were within normal limits for some time after surgery, but began to show an increase, while serum carcinoembryonic antigen, CA19-9, and CA-125 levels stayed normal from preoperative period. Pulmonary recurrence was diagnosed eventually. The elevated serum AFP level was the first indication of disease recurrence in this patient.

For further accrual of cases, we proposed the diagnostic criteria for AFP+ EC in Table 4. The relationship between histomorphology, immunohistochemistry, and serum AFP levels is not straight-forward in gastric and pulmonary cancers and they do not always concur. In WHO Classifications of respective fields, histological classification is always given priority. Fetal adenocarcinoma of the lung is defined by histomorphology and only a few immunohistochemistry to differentiate between low-grade and high-grade subtypes (β-catenin) and to exclude endometriosis (TTF1 positivity for low-grade fetal adenocarcinoma) (51). Hepatoid adenocarcinoma of the lung is mentioned, but not formally defined (51). AFP-producing carcinoma, hepatoid adenocarcinoma, and adenocarcinoma with enteroblastic differentiation of the stomach are also not formally defined, but hepatoid and enteroblastic adenocarcinomas seem to be histomorphological categories, while AFP-producing carcinoma seems to denote a loose collection of carcinomas with immunohistochemical or serological evidence of AFP production (52). Definition employing IHC has been used in some studies: Kinjo et al. defined AFP-producing gastric cancer as gastric tumors with immunohistochemical positivity for AFP or glypican 3 in ≥ 1% of tumor cells (47); Akazawa et al. defined gastric adenocarcinoma with enteroblastic differentiation as a morphologically appropriate tumor that stains positive for AFP, glypican 3, or SALL4 in > 10% of the tumor (3); Fujimoto et al. used somewhat different thresholds for the latter tumor and required that > 5% of the tumor be positive for glypican 3 or SALL4 or that > 1% of the tumor be positive for AFP (49). Our proposed diagnostic criteria are largely in line with these definitions and prioritize histomorphology with discreet use of immunohistochemistry. Elevation of serum AFP levels are treated as important, but nonessential characteristics. To date, no cases of endometrial fetal gut-like or hepatoid carcinoma without elevated serum AFP levels have been reported, but this may be due to publication bias. Since we did not make elevated serum AFP levels a prerequisite for diagnosis, cases with appropriate morphology and immunophenotype but normal serum AFP levels can be diagnosed as AFP+ EC with these criteria. This decision would also allow for the diagnosis of cases for which serum AFP values are not available (our cases 1, 2, and 4).

**Table 4:** Proposed essential and desirable diagnostic criteria for AFP-producing endometrial carcinomas.

	**Essential**	**Desirable**
AFP-producing endometrial carcinoma	- Histomorphology consistent with carcinoma. Hepatoid morphology is allowed. - Positive IHC for AFP in ≥ 1% of tumor cells. - Absence of morphological patterns characteristic of yolk sac tumor but inconsistent with carcinoma. These include a reticular/microcystic pattern, Schiller-Duval bodies, and loose myxoid stroma.	- Positive IHC for SALL4. - Negative or non-diffuse IHC for ER, PR, PAX8 and CK7.
Hepatoid carcinoma of the endometrium	- Carcinoma resembling hepatocellular carcinoma.	- Positive IHC for AFP and/or SALL4. - Negative or non-diffuse IHC for ER, PR, PAX8 and CK7.
Fetal gut-like carcinoma of the endometrium	- Carcinoma resembling fetal gut epithelium (columnar epithelium with clear cytoplasm). - Exclusion of clear cell carcinoma and secretory variant of endometrioid carcinoma	- Positive IHC for AFP and/or SALL4. - Negative or non-diffuse IHC for ER, PR, PAX8 and CK7.
Non-clear glandular AFP-producing endometrial carcinoma	- Gland-forming adenocarcinoma without clear cells. - Positive IHC for AFP and/or SALL4.	- Negative or non-diffuse IHC for ER, PR, PAX8 and CK7.

*AFP, α-fetoprotein; CK, cytokeratin; ER, estrogen receptor; IHC, immunohistochemistry; PR, progesterone receptor*.

Differential diagnoses of AFP+ EC include endometrioid, clear cell, serous, and gastric-type uterine carcinoma, and metastatic carcinoma. Some reports in the previous literature have emphasized the similarity between AFP+ EC and secretory variant of endometrioid carcinoma, both being composed of tall columnar cells with clear cytoplasms (Figures 2A,B) (32, 53). Since AFP+ EC can grow in solid, papillary, and glandular patterns with variable amounts of optically clear cells and hobnail-like cells and is positive for HNF1β on IHC at least weakly, clear cell carcinoma also looms as a serious diagnostic consideration (30). Incidentally, HNF1β, which is widely known as a positive marker of clear cell carcinoma of the gynecological tract, also stains positive in yolk sac tumor (54). In this context, immunostaining with napsin A would be helpful, as it would be positive in clear cell carcinoma and negative in AFP+ EC (30, 32). Non-clear glandular AFP+ EC (Figure 4A) and hepatoid carcinoma (Figure 3) might resemble low-grade and high-grade endometrioid carcinoma, respectively. High-grade cytology and abnormal p53 IHC can lead to the consideration of serous carcinoma. If AFP+ EC extends to the uterine cervix, clear cytoplasms might cause confusion with gastric-type adenocarcinoma. Although not explored in our study, glandular yolk sac tumor is reported to be positive for CDX2, reflecting its endodermal differentiation (34); this can be a cause of misdiagnosis as metastatic gastrointestinal adenocarcinoma, especially in patients with a history of these malignancies (55). In all cases, a low threshold to perform relevant immunostaining and evaluation of serum AFP levels can be a key to correct diagnoses. Immunohistochemical characteristics of AFP+ EC and its differential diagnoses are summarized in Table 5. Regarding cervical carcinomas, it should be noted that AFP-producing cervical carcinoma with fetal gut-like morphology has been reported (45, 46).

**Table 5:** Immunohistochemical characteristics of AFP-producing endometrial carcinoma and other endometrial neoplasms in differential diagnosis.

**Tumor type**	**AFP**	**SALL4**	**PAX8**	**CK7**	**ER/PR**	**HNF1β**	**Napsin A**	**p53**	**Others**
AFP-producing endometrial carcinoma	+	+	-	-/+	-	+	-	Aberrant/wt	
Endometrioid carcinoma	-	-	+	+	+	-	-	wt	
Clear cell carcinoma	-	-	+	+	-	+	+	wt/aberrant	
Serous carcinoma	-	-	+	+	-/+	-	-	Aberrant	
Yolk sac tumor^a^	+	+	-	-	-	+	-	Variable (32)	
Gastric-type adenocarcinoma of uterine cervix	-	-	+	+	-	+	-	Aberrant/wt	HIK1083+, MUC6+
AFP-producing adenocarcinoma of uterine cervix (45,46)	+	+	-	NK	-	NK	NK	Aberrant in 2 reported cases	
Metastatic adenocarcinoma	-	-	Variable	Variable	Variable	Variable	Variable	Variable	

*AFP, α-fetoprotein; CK, cytokeratin; ER, estrogen receptor; NK, not known; PR, progesterone receptor; wt, wild-type*.

a *There is a conceptual overlap between AFP-producing endometrial carcinoma and yolk sac tumor*.

Some observations are warranted regarding the pathogenesis of AFP+ EC. This subset of endometrial carcinoma is unusual in that it is immunohistochemically negative for PAX8 and thus cannot be said to show Müllerian differentiation. However, many cases are associated with Müllerian carcinoma or carcinosarcoma. Although not directly comparable to AFP+ EC, Acosta et al. (33) showed that in malignant tumors of the uterus and ovaries with Müllerian and germ cell components, both components are clonally related. We believe it reasonable to suppose that AFP+ EC is of Müllerian origin and exhibits divergent line of differentiation. Another example of endometrial carcinoma of Müllerian origin with divergent differentiation is endometrial mesonephric-like adenocarcinoma (56).

Whether AFP+ EC and “AFP-producing carcinomas” of other organs such as stomach and lung should be classified as “carcinomas” or “yolk sac tumors” is an unresolved nosological problem. Several arguments can be put forward for the case of separation of AFP+ EC from yolk sac tumor. One important consideration would be the problem of case recognition. It is quite understandable that some pathologists would be reluctant to diagnose such cases as are reported here as a yolk sac tumor. For others, a diagnosis of yolk sac tumor might not be on the differential list at all. Once the existence of endometrial carcinoma with AFP production is widely recognized, this highly malignant tumor will be more frequently diagnosed appropriately. Second, there is the issue of continuity with previous literature: before allowing the concept of somatically derived yolk sac tumor to encompass all AFP-producing neoplasms of older patients aside from liver cancer, how to position cases reported in the past as AFP-producing carcinomas should be considered. Third, recognizing the category of AFP+ EC would make the classification of AFP-producing endometrial neoplasms more consistent with classifications of tumors in other organs, such as stomach and lung. Whether this separation is justified or not should be examined with the accumulation of further cases. From the existing data, the frequency of *TP53* abnormalities seems to be higher in AFP+ EC than in cases reported as somatically derived yolk sac tumors of the endometrium (32).

Our study has several limitations. First, because AFP+ EC is a rare tumor, we could only study a limited number of cases in detail. To compensate for this, we included cases from the previous literature in some parts of the analysis, which may have resulted in a heterogeneous set of cases. Second, we could not identify any endometrial yolk sac tumors morphologically comparable to yolk sac tumors in younger patients in our predesignated time period: comparing them with AFP+ ECs should have enabled us to study whether AFP+ EC is better considered a “carcinoma,” “germ cell tumor,” or something intermediate. These points should be addressed in future studies and shed some light on these aggressive neoplasms.

In conclusion, we revealed that AFP+ EC is a clinicopathologically distinct subtype of endometrial carcinoma, occurring in 1.8% of endometrial carcinoma cases. They occur mainly in postmenopausal women and are associated with *TP53* abnormalities and vascular infiltration, which can be extensive. These carcinomas can show aggressive behavior even in FIGO stage I cases and merit further studies to elucidate their characteristics and optimal treatment.

## Data Availability Statement

The original contributions presented in the study are included in the article/Supplementary Materials, further inquiries can be directed to the corresponding author.

## Ethics Statement

The studies involving human participants were reviewed and approved by Institutional Review Board of Kindai University Faculty of Medicine. The patients/participants provided their written informed consent to participate in this study.

## Author Contributions

TO drafted the manuscript. TO and AI conceived the study, performed histopathological analysis, and analyzed data. KM participated in the study design. TO and NS performed molecular analysis. MH provided technical assistance. KM, MM, TS, and NM provided care for the patients and collected clinical data and specimens. All authors revised the manuscript.

## Conflict of Interest

The authors declare that the research was conducted in the absence of any commercial or financial relationships that could be construed as a potential conflict of interest.

## Publisher's Note

All claims expressed in this article are solely those of the authors and do not necessarily represent those of their affiliated organizations, or those of the publisher, the editors and the reviewers. Any product that may be evaluated in this article, or claim that may be made by its manufacturer, is not guaranteed or endorsed by the publisher.

## Abbreviations

AFP: α-fetoprotein
AFP+ EC: α-fetoprotein-producing endometrial carcinoma
FFPE: formalin-fixed paraffin-embedded
FIGO: International Federation of Gynecology and Obstetrics
IHC: immunohistochemistry.
