# Supplementary material for: α-Fetoprotein-Producing Endometrial Carcinoma Is Associated With Fetal Gut-Like and/or Hepatoid Morphology, Lymphovascular Infiltration, TP53 Abnormalities, and Poor Prognosis: Five Cases and Literature Review
Source: Front Med (Lausanne). 2021 Dec 15;8:799163. doi: 10.3389/fmed.2021.799163 (PMC8714782; doi:10.3389/fmed.2021.799163)
Supplement: Supplementary file 1 [file Data_Sheet_1.docx]

Supplementary Material

**Supplementary Table 1. Antibodies used for immunohistochemistry**

| Antibody | Clone | Dilution | Source |
| --- | --- | --- | --- |
| AFP | polyclonal | working solution | Roche, Bazel, Switzerland |
| SALL4 | 6E3 | 1:500 | Abnova Corporation, Taipei, Taiwan |
| PAX8 | polyclonal | 1:1000 | ProteinTech Group, Chicago, IL |
| CK7 | OV-TL12/30 | 1:800 | Dako, Santa Clara, CA |
| ER | SP1 | working solution | Roche |
| PR | 1E2 | working solution | Roche |
| HNF1β | polyclonal | 1:500 | Atlas Antibodies, Stockholm, Sweden |
| Napsin A | polyclonal | working solution | Nichirei Biosciences, Tokyo, Japan |
| p53 | DO-7 | 1:50 | Dako |
| MLH1 | M1 | working solution | Roche |
| PMS2 | A16-4 | working solution | Roche |
| MSH2 | G219-1129 | working solution | Roche |
| MSH6 | SP93 | working solution | Roche |
| HER2 | 4B5 | working solution | Roche |
| D2-40 | D2-40 | 1:40 | Dako |
| CD31 | JC70A | 1:50 | Leica Biosystems, Nussloch, Germany |

**Supplementary Table 2. Primers used for PCR amplification and sequencing**

| Gene | Exon | Sequence |
| --- | --- | --- |
| TP53 | exon 4 | 5′-TTGCTGCCGTCTTCCAGTTGCT-3′ |
|  |  | 5′-GAGGGGCCAGACCTAAGAGCAA-3′ |
|  | exon 5 | 5′-GCTCAGATAGCGATGGTGAGCA-3′ |
|  |  | 5′-CCTTAGCCTCTGTAAGCTTCAGTT-3′ |
|  | exon 6 | 5′-TGCTTGCCACAGGTCTCCCCAA-3′ |
|  |  | 5′-GGATGTGATGAGAGGTGGATGGGT-3′ |
|  | exon 7 | 5′-TTGGGAGTAGATGGAGCCTGGT-3′ |
|  |  | 5′-TGGTGTTGTTGGGCAGTGCTAG-3′ |
|  | exon 8 | 5′-TCCGCAAGAAAGGGGAGCCTCA-3′ |
|  |  | 5′-TGATGGCAAATGCCCCAATTGCA-3′ |
| POLE | exon 9 | 5′-GTGGGTGTTCAGGGAGGCCTAAT-3′ |
|  |  | 5′-CCATCCCAGGAGCTTACTTCCCA-3′ |
|  | exon 13 | 5′-TTTGCCAGTTCTCAGGGGTTCC-3′ |
|  |  | 5′-CACAGTAAGGAGACCGGCACAG-3′ |
|  | exon 14 | 5′-TGTCCTGTGCCGGTCTCCTTAC-3′ |
|  |  | 5′-ACCTCCATTCAGCTCCAGTGCAT-3′ |
| CTNNB1 | exon 3 | 5′-CACTGAGCTAACCCTGGCTATCA-3′ |
|  |  | 5′-CCAGCTACTTGTTCTTGAGTGAAGGACTG-3′ |
| KRAS (39) | exon 2 | 5′-GAATGGTCCTGCACCAGTAA-3′ |
|  |  | 5′-GTGTGACATGTTCTAATATAGTCA-3′ |
| PIK3CA (40) | exon 10 | 5′-TTGAAAATGTATTTGCTTTTTCTGT-3′ |
|  |  | 5′-CATGTAAATTCTGCTTTATTTATTCCA-3′ |
|  | exon 21 | 5′-CATTTGCTCCAAACTGACCA-3′ |
|  |  | 5′-GGTCTTTGCCTGCTGAGAGT-3′ |
